# Supplementary material for: Bioavailability of Lutein from Marigold Flowers (Free vs. Ester Forms): A Randomised Cross-Over Study to Assess Serum Response and Visual Contrast Threshold in Adults
Source: Nutrients. 2024 May 8;16(10):1415. doi: 10.3390/nu16101415 (PMC11123982; doi:10.3390/nu16101415)

Figure S1. Correlations between serum lutein concentration (µg/dL, X axis) and CT (Y axis) in the total group (A), in the group aged 20-35 y (B) and 50-65 y (C). Line colours represent frequencies: high (red), medium (green) and low (blue).

A) Total group

Basal, with glare

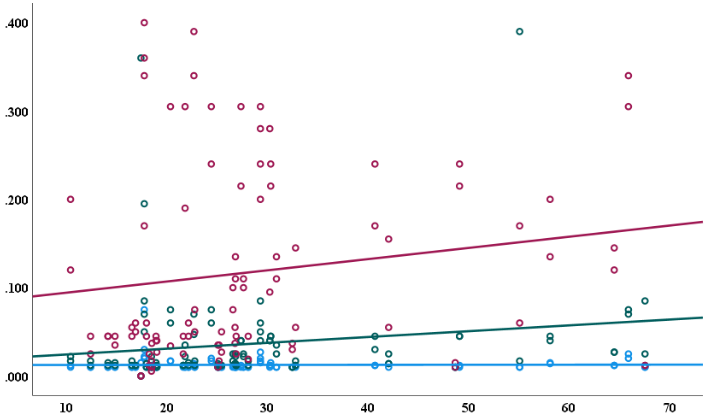

60 d, with glare

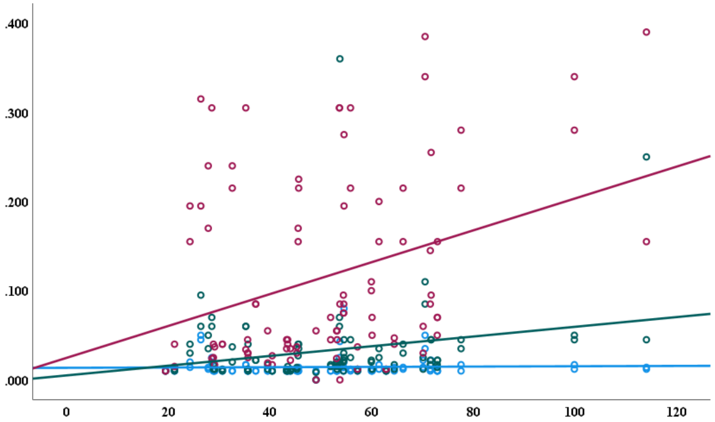

Basal, without glare

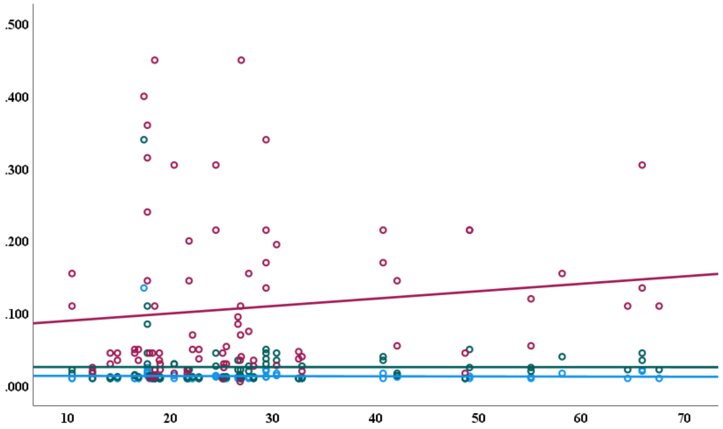

60 d, without glare

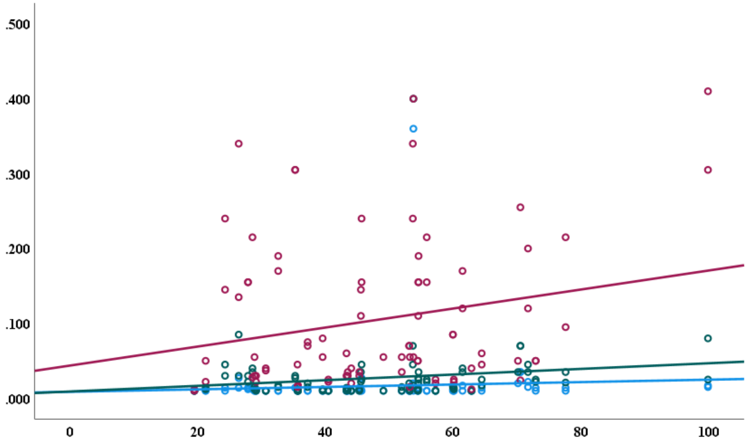

B) group 20-35 y.

Basal, with glare

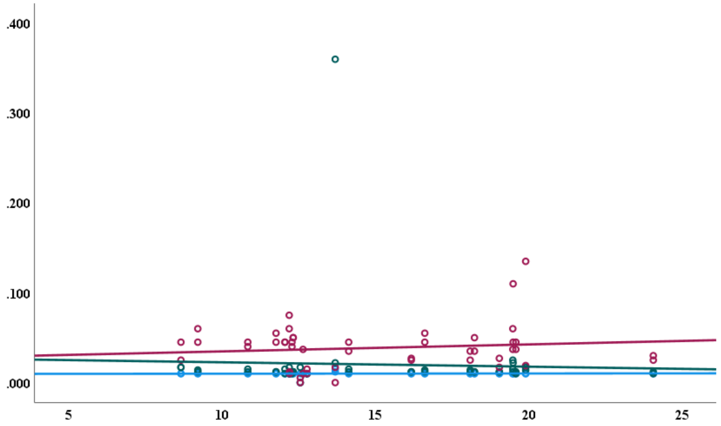

60 d, with glare

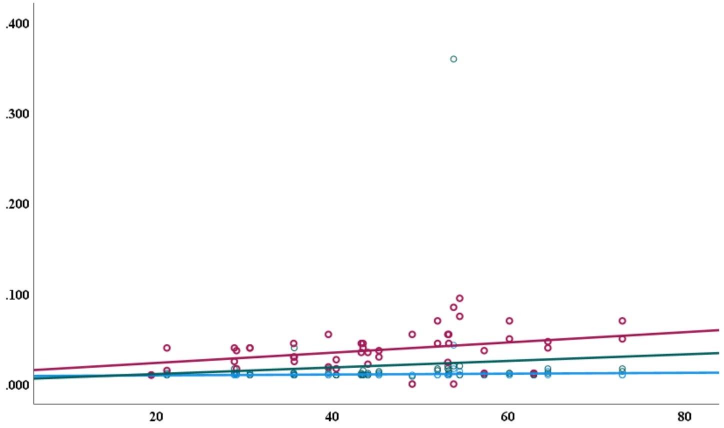

Basal, without glare

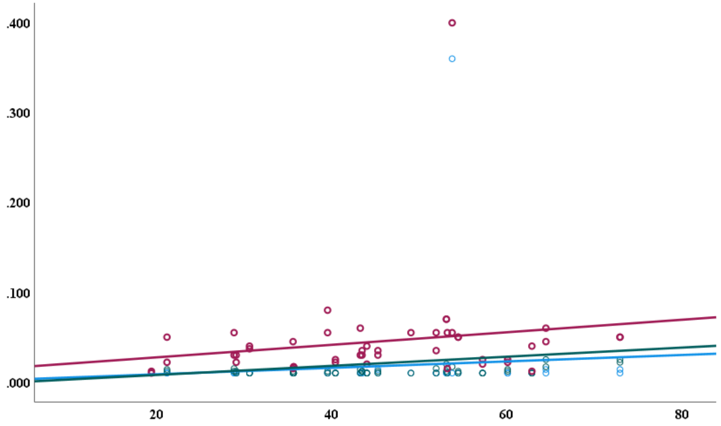

60 d, without glare

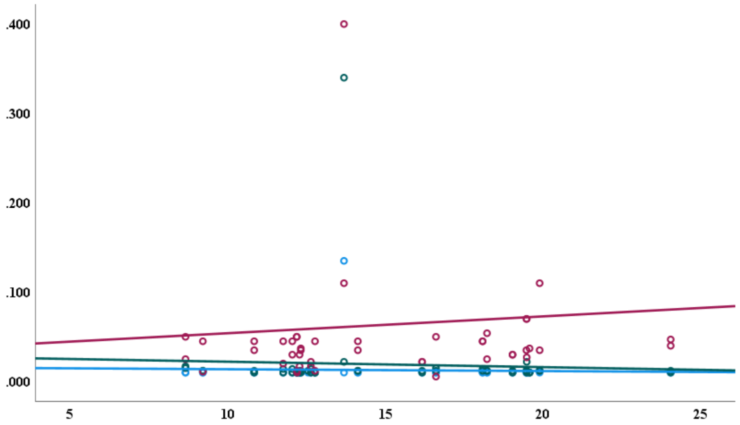

C) group 50 – 65 y.

Basal, with glare

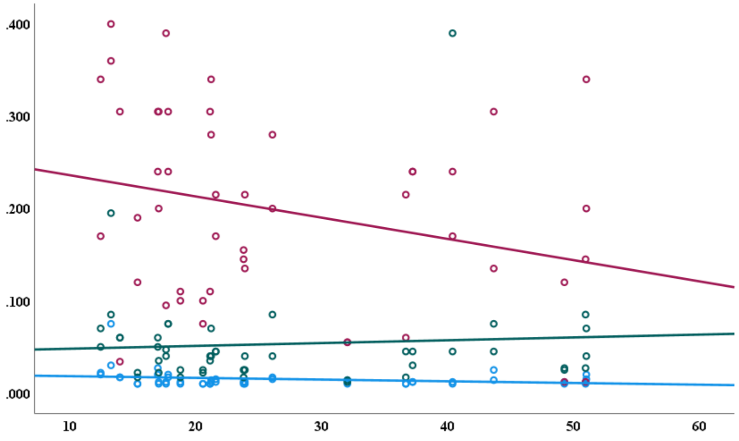

60 d, with glare

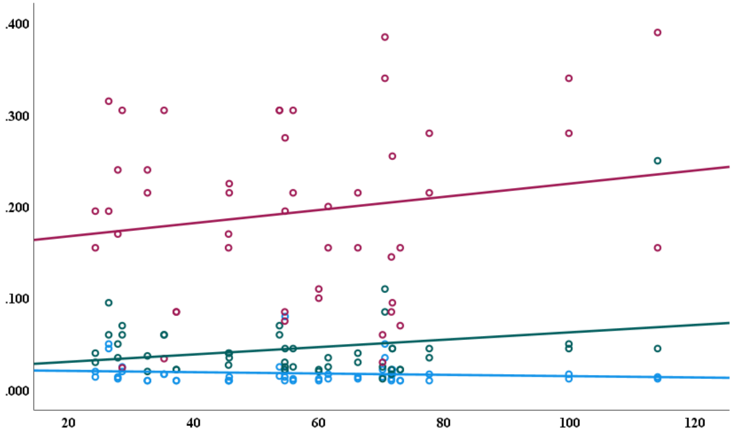

Basal, without glare

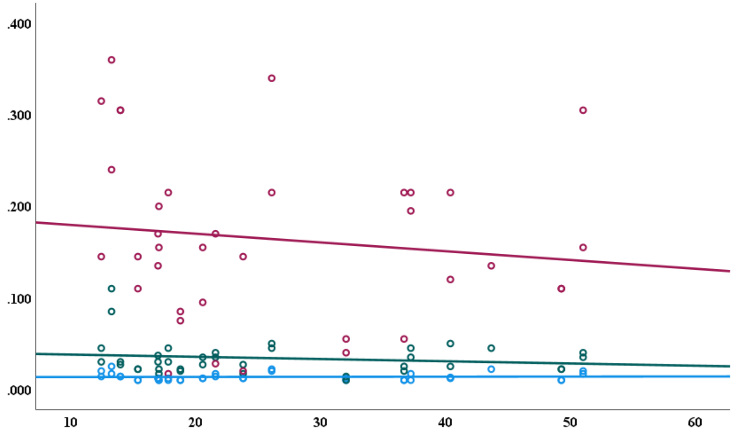

60 d, without glare

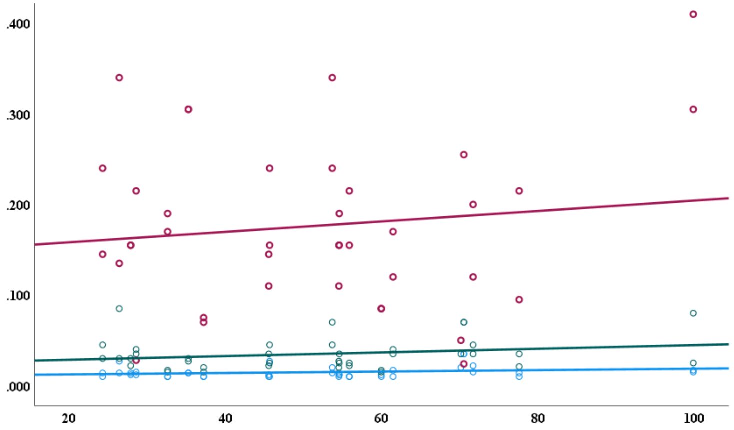

Supplement: Supplementary file 1 [file nutrients-16-01415-s001.zip › nutrients-2906935-supplementary.pdf]
